# 

**Published:** 1881-04

**Authors:** 


Vol. II.] TWO DOLLARS A YEAR. SINGLE COPIES 50 CENTS. [No. 2.

### THE JOURNAL

(FORMERLY ARCHIVES)

OF

### COMPARATIVE MEDICINE AND SURGERY.

A Quarterly Journal

OF THE

ANATOMY, PATHOLOGY, AND THERAPEUTICS OF THE LOWER ANIMALS.

NEW YORK:
W. L. HYDE & CO., PUBLISHERS, NO. 22 UNION SQUARE.
1881.

